# Anti-Obesity Effect of *Dictyophora indusiata* Mushroom Polysaccharide (DIP) in High Fat Diet-Induced Obesity *via* Regulating Inflammatory Cascades and Intestinal Microbiome

**DOI:** 10.3389/fendo.2020.558874

**Published:** 2020-11-19

**Authors:** Sadia Kanwal, Shams Aliya, Yi Xin

**Affiliations:** ^1^ Department of Biotechnology, College of Basic Medical Sciences, Dalian Medical University, Dalian, China; ^2^ Faculty of Life Sciences, Institute of Cancer Therapeutics, University of Bradford, Bradford, United Kingdom

**Keywords:** obesity, high-fat diet, gut microbiota, inflammation, *Dictyophora indusiata* polysaccharide (DIP)

## Abstract

Obesity is a multifactorial metabolic disorder characterized by low-grade chronic inflammation, hyper-permeability of the gut epithelium, and perturbation of the intestinal microbiome. Despite the numerous therapeutic efficacies of *Dictyophora indusiata* mushroom, its biological activity in alleviating obesity through regulation of the gut microbiota and inflammatory cascades remain obscure. Henceforth, we determined the modulatory impact of *D. indusiata* polysaccharide (DIP) in the high-fat diet (HFD)-induced obesity mice model. The experimental subjects (BALB/C mice) were supplemented with chow diet (Control group), high-fat diet (HFD group), or HFD along with DIP at a low dose [HFD + DIP(L)] and high dose [HFD + DIP(H)]. Obesity-related parameters, including body weight gain, epididymal adipocyte size, fat accumulation, adipogenic markers, lipogenic markers, inflammatory associated markers, intestinal integrity, and intestinal microbiome, were elucidated. Our findings demonstrated that the oral administration of DIP at low dose partially and at high dose significantly reversed HFD-induced obesity parameters. Furthermore, the body weight, fat accumulation, adipocyte size, adipogenic and liver associated markers, glucose levels, inflammatory cytokines, and endotoxin (Lipopolysaccharide, LPS) levels were reduced considerably. Moreover, the study revealed that DIP treatment reversed the dynamic alterations of the gut microbiome community by decreasing the Firmicutes to Bacteroidetes ratio. These findings led us to infer the therapeutic potential of DIP in alleviating HFD-induced obesity *via* regulating inflammatory cascades, modulating intestinal integrity and intestinal microbiome community.

## Introduction

Obesity is one of the major health concerns across the world that has been implicated with multiple health problems and a reduced life expectancy (1, 2). The prevalence of obesity has been strikingly increased in recent years, and it has been estimated that by 2030 obesity rate will reach up to 20% of the adult population (3). Approximately 500 million individuals are obese, and 1.4 billion are overweight globally (4). Obesity is characterized by excessive fat accumulation due to various factors, including genetic predisposition, high-calorie energy intake, and sedentary lifestyle (5). Obesity predisposes an individual to develop and aggravate a broad spectrum of disorders, including type 2 diabetes, insulin resistance, fatty liver disease, gastrointestinal problems, cardiovascular disease, respiratory problems, and various kinds of cancers (6–9). Considering the adverse impact of obesity on individual health, it is of great challenge to reduce and halt obesity in the modern world.

A growing body of evidence demonstrates that a common trait identified in obese humans and animal studies is the hyper permeable gut (10, 11). A leaky gut eventually causes local or systemic inflammation due to increase susceptibility to antigens leading to impairment of gut barrier function, reduced tight junction protein (TJP) expression, and increased intestinal permeability to pathogenic microorganism (12–15). Moreover, high fat-induced obesity enhances lipopolysaccharide (LPS) levels in the bloodstream, ultimately causing inflammation and insulin resistance (13, 16, 17). LPS is the main component in synthesizing the outer membrane of gram-negative bacteria that triggers several transcription factors associated with inflammation resulting in intestinal barrier damage (18). Furthermore, many reports have shown enhanced pro-inflammatory cytokine production, such as tumor necrosis factor-α (TNF-α), interleukin (IL)-1 β, IL-6, and reduced anti-inflammatory cytokine secretion in obese animal models (19, 20). Another study shows that a higher level of cytokines triggers not only chronic inflammation but also insulin resistance (7).

The role of the gut microbiota in the predisposition of obesity and related disorders is well recognized (21–23). The intestinal flora maintains the host physiology *via* harnessing energy from complex and indigestible dietary compounds such as polysaccharides and polyphenols (24–27). The alteration of bacterial composition leads to a condition known as gut dysbiosis (28–30). Gut microbiome perturbation (dysbiosis) has been implicated with several human ailments, including inflammatory diseases, obesity, metabolic, and neurological disorders (31–35). High fat dietary intake is associated with the alteration of the composition of the intestinal microbiota community, distinguished by reduced bacterial diversity and richness, and increase abundance of Firmicutes to Bacteroidetes ratio (15, 36–41). Various *in-vivo* studies have demonstrated that gut dysbiosis is a major contributing factor in obesity and type 2 diabetes (42–44). Furthermore, clinical studies have also proven the association of intestinal dysbiosis in the etiology of obesity and obesity-related disorders (45). Thus the dynamic shift of intestinal flora due to high-fat diet consumption not only develops obesity but also disrupts intestinal integrity and increases endotoxemia (LPS) levels (16). The enhanced LPS levels eventually trigger inflammatory cascades and enhance insulin resistance due to the activation of inflammatory signaling pathways (13, 17, 46, 47). Nevertheless, the modulation of gut microbiome through dietary interventions, including herbal plants, probiotics/prebiotics, is promising strategies to reduce inflammatory-related problems such as obesity and associated metabolic syndrome (48–53).

Several drugs have been developed in recent years to combat obesity and obesity-related health issues. Nonetheless, long term dependency on anti-obesity drugs has been implicated with severe side effects and weight regain upon discontinuation (54–56). Henceforth researchers are focusing on finding novel therapeutic arsenals to prevent this social and economic catastrophe and overcome obesity-associated problems. In this regard, the role of natural dietary compounds such as mushroom polysaccharides in treating multiple diseases is drastically increasing, owing to the plethora of health ameliorating properties. The prebiotic sources such as mushroom polysaccharides that serve as a substrate for microbes residing in the gastrointestinal tract enhance the growth of microbes that exert a beneficial physiological impact on host immunity (57–60). Additionally, dietary fiber encourages the growth of short-chain fatty acids’ (SCFAs’) producing bacteria that inhibit inflammatory reactions (15, 58, 61, 62). Moreover, prebiotics not only exerts a beneficial impact on the gut microbial community but also modulates gut barrier integrity *via* regulating TJP expression and reducing endotoxin levels in the bloodstream (63).

Accumulated pieces of evidence suggest that *Dictyophora indusiata* mushroom derived polysaccharides possess a variety of biological activities (64) such as anti-inflammatory, anti-tumor, antioxidant, and immunomodulatory activities (65–68). In our previous studies, we have reported the modulatory effect of *Dictyophora indusiata* polysaccharide (DIP) in antibiotic driven intestinal dysbiosis and DSS induced ulcerative colitis (69, 70). The anti-obesity effect of DIP *via* regulation of obesity-associated parameters and improving gut microbiome community is yet to be explored. Given the protective effect of *Dictyophora indusiata* mushroom in combating various health problems, in the present study, we further investigated the effect of DIP on HFD-induced obesity. Our findings revealed that HFD-induced obesity was reduced upon DIP supplementation. The intestinal integrity and inflammatory reactions were ameliorated. Moreover, the gut microbiome shift was reversed. These results thus demonstrate that the *D. indusiata* derived polysaccharide (DIP) may be used as a potential prebiotic compound in treating obesity and associated issues.

## Materials and Methods

### Experimental Subjects and Housing

**Figure 1:**
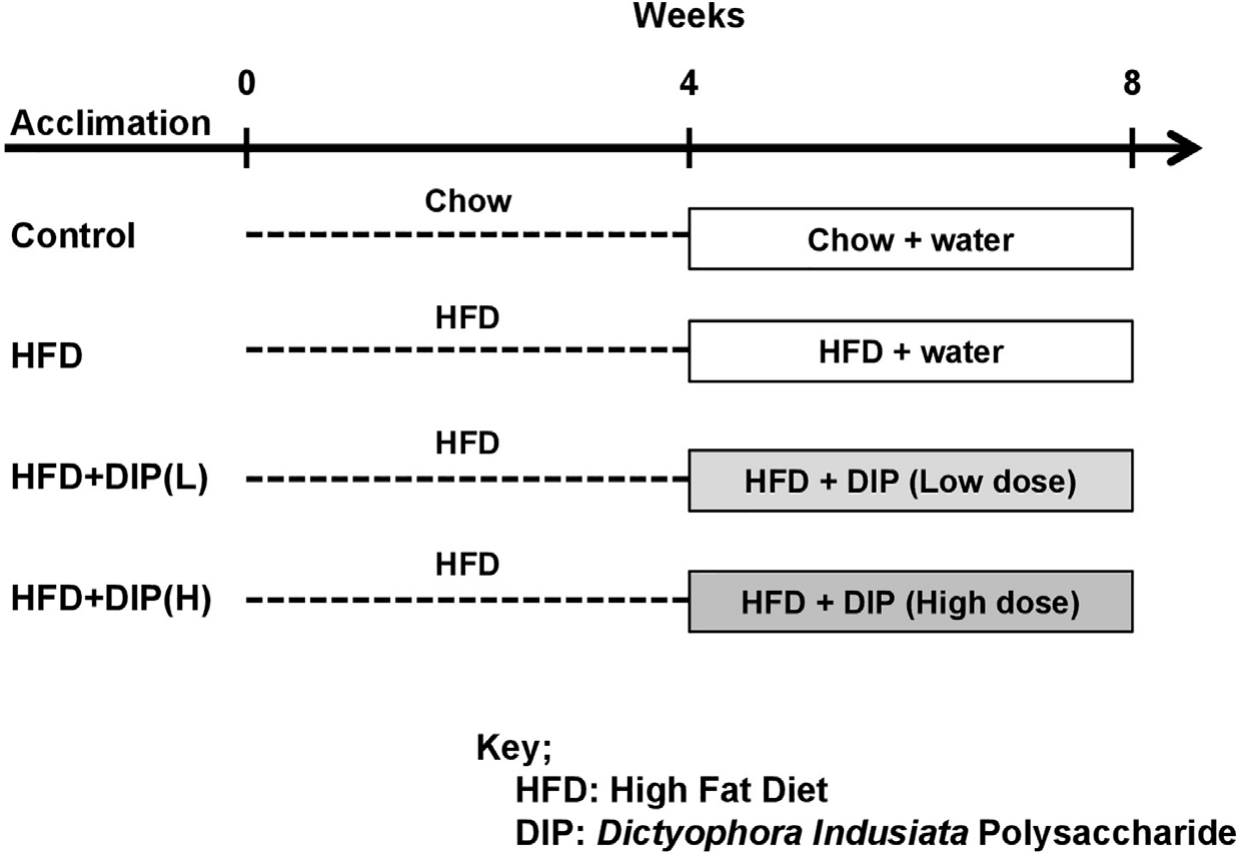
Experimental design. Experimental mice were categorized into four groups (n = 8 each group). Control or vehicle group, HFD group, HFD followed by DIP(L) group and HFD followed by DIP(H) group.

Specific-pathogen-free (SPF) level inbred male BALB/c mice aged seven weeks old with similar initial body weight (20 ± 3 g) approved by the Animal Care and Research Ethics Committee of Dalian Medical University, China (Approval Number: SYXK 2016–2018) were randomly assigned to four groups (eight mice per group). All the animals used in the study were acclimated for a period of one week prior to experimentation. All experimental procedures were approved by the ethics committee of Dalian Medical University. The experimental subjects of the respective group were housed randomly in two separate cages (four mice per cage, so two separate cages for each group). All the cages were maintained in an environmentally controlled room (20–22°C) temperature and 55 ± 5% relative humidity with 12–12 h light/dark cycle). The experimental design is presented in **Figure 1**. The mice were placed on standard commercial chow diet (control group) or 60% high-fat diet (HFD groups) (Jiangsu Medison Biomedical Co., Ltd., Yangzhou, Jiangsu Province, China) for 8 weeks. On 4^th^ week, the chow and HFD group were gavaged with water (200 µl). The crude polysaccharide (DIP) from *D. indusiata* was administered (200 µl) daily at a low dose [HFD + DIP(L) group] of 200 mg/kg body weight and a high dose [HFD+DIP(H) group] of 400 mg/kg body weight *via* oral gavage from week 4^th^ till the 8^th^ week. The dosage and treatment duration are based upon a previous study (71). The bodyweight of each subject from the respective group was monitored once a week, and food intake was measured every day in the morning. After 8 weeks, all the experimental subjects were sacrificed. The organs, tissues, and blood samples were collected for the subsequent analysis.

### Antibodies, Kits, and Reagents

The fruiting body of the mushroom *D. indusiata* was obtained from Anhui Joy Lok Food Co., Ltd., Ningde, Fujian Province, China. The DNA extraction kit (QIAamp DNA Stool Mini Kit) and gel purification kit (Agencourt AMPure XP 60 mL Kit) were from Qiagen (Hilden, Germany) and Beckman Coulter (Brea, CA, USA), respectively. The primary antibodies β-actin, claudin-1, occludin, zonula occludens-1 (ZO-1), secondary antibodies, and the Radioimmunoprecipitation assay (RIPA) buffer were from Proteintech (Wuhan, China). The horseradish peroxidase-conjugated secondary antibody was from ZSGB-BIO (Beijing, China). The bicinchoninic acid (BCA) protein assay kit was from Pierce Rockford, IL, USA. Polyvinylidene difluoride (PVDF) membranes were from Immobilon TM-P, Millipore, Massachusetts, USA, and WesternBright™ ECL substrate was from Advansta, Inc., Menlo Park, CA, USA. The ELISA kits were purchased from Shang Hai Lengton Bioscience Co, Ltd., Shanghai, China. All the other chemicals used in this study were of analytical grade and purchased from standard commercial sources.

### Extraction and Chemical Analysis of Crude Polysaccharide DIP From the Mushroom *D. indusiata*

The polysaccharide extraction method, chemical analysis, and monosaccharide composition are described in our previous study (69). Briefly, the sugar content and crude polysaccharide were determined with bicinchoninic acid (BCA) and phenol sulfuric acid method. The monosaccharide composition was determined with high-performance liquid chromatography (HPLC). The polysaccharide yield was 13.2%, and the main functional components were glucose 59.84%, mannose 23.55%, and galactose 12.95%.

### Morphometry Analysis of Adipose Tissues

Histological analysis was performed to determine the size of adipose tissue. Freshly isolated epididymal adipose tissues from all the experimental groups were fixed overnight in 10% formalin. The tissue sections were deparaffinized and embedded in paraffin, followed by sectioning. 5 μm paraffin sections were stained with hematoxylin and eosin, and images were obtained using a microscope (Leica Microsystems, Wetzlar, Germany). For the measurement of adipocytes, the number of adipocytes was counted for each sample from the respective group, and the average adipocyte size was analyzed using the Image J software (National Institutes of Health, USA) as described previously (72).

### Determination of Blood Insulin and Glucose

Blood insulin levels of overnight fasted (12 h) mice were measured using a commercial ELISA kit (Wuhan Cusabio Biotech Co., Ltd., China) according to the manufacturer’s recommendations. Blood glucose of overnight fasted (12 h) mice was determined with a glucose meter (Roche Diagnostics, Switzerland).

### Biochemical Analysis and Cytokine Level Measurement

Blood samples were centrifuged at 3,000× g for 10 min, and serum was separated to evaluate serum endotoxin (lipopolysaccharide, LPS) levels using a commercial kit based on the guidelines provided by the supplier (LAL, Limulus amebocyte lysate assay; Chinese Horseshoe Crab Reagent Manufactory Co., Ltd. Xiamen, China). The pro-inflammatory cytokine levels including tumor necrosis factor-alpha (TNF-α), interleukin 6 (IL-6), interleukin 1β (IL-1β), and anti-inflammatory cytokines such as interleukin 4 (IL-4) and interleukin 10 (IL-10) in the serum were determined using a mouse ELISA kit (Shang Hai Lengton Bioscience Co., Ltd.) Shanghai, as per manufacturer’s instructions. Alanine transaminase (ALT), aspartate transaminase (AST), total cholesterol (TC), and triglyceride (TG) levels were estimated using a chemical analyzer (Hitachi, Tokyo, Japan). Serum free fatty acids (FFAs) were measured using a commercial kit (Biovision, USA).

### RNA Extraction and Quantitative Real-Time Reverse-Transcription PCR

The RNA was extracted using TRIzol reagent (Invitrogen Life Technology Gaithersburg, MD, USA) according to the manufacturers’ instructions. Quantitative real-time reverse-transcription PCR (qRT–PCR) was performed in triplicate using SYBR Green (Kapa SYBR Fast Master Mix) and Real-Time PCR thermocycler (Applied Biosystems StepOnePlus™). The total volume of the reaction mixture for each tube was 10 µl containing 1.5 µl of cDNA, 1 µl of target primers, 2.5 µl of nuclease-free water, and 5 µl of SYBR Green Master Mix. RT-PCR was performed for 50 cycles, and PCR conditions were as follows: Initial pre-incubation at 95°C for 10 min, denaturation for 15 s at 94°C, annealing for 30 s at 60°C followed by elongation for 30 s at 72°C. The relative gene expression was quantified by using the 2^–ΔΔCt^ method (73). GAPDH was used as an internal control, and all the reactions were performed in triplicate. The primer sequences used in the study are shown in **Supplementary Table S1**.

### Immunoblotting Assay

Intestinal tissue samples (100 mg) were homogenized using RIPA lysis buffer containing a protease inhibitor (Transgene Biotech, Beijing, China) and then centrifuged at 12,000 g for 5 min at 4°C. The supernatant containing protein was removed from the pellet, and the protein concentration was determined using the BCA protein assay kit (Pierce, Rockford, IL, USA) according to the manufacturer’s instructions. 10% sodium dodecyl sulfate-polyacrylamide gel (SDS PAGE) was employed for fractionation of protein lysates followed by blotting onto Polyvinylidene difluoride (PVDF) membrane. 5% non-fat milk in TBST was used for blocking non-specific binding for 1 h at room temperature. After blocking, membranes were probed overnight at 4°C with primary antibodies including *β*-actin (catalog no. 20536-1-AP, 1:2000), claudin-1 (catalog no. 13050-1-AP, 1:500), occludin (catalog no. 13409-1-AP, 1:2000), and ZO-1 (catalog no. 21773-1-AP, 1:500). Membranes were then incubated with horseradish peroxidase-conjugated secondary antibody (IgG) (catalog no. SA00001-2, 1:6,000) for 1 h at room temperature. Protein bands were developed with WesternBright™ ECL substrate (Advansta, Inc., Menlo Park, CA, USA), and images were captured by ChemoDocTM XRS + Imager-Bio-Rad (Bio-Rad Laboratories, Inc., Hercules, CA, USA).

### DNA Extraction and 16S rRNA Gene Amplification (Illumina MiSeq Sequencing)

Metagenomic DNA extraction, 16S rRNA gene amplification, and sequencing were executed as described in our previous study (69). Briefly, the 16S rRNA gene V4 region was amplified from genomic DNA using the universal bacterial primers: (515F, 5′-GTGCCAGCMGCCGCGGTAA-3′ and 806R, 5′-GGACTACHVGGGTWTCTAAT-3′). The amplicons were normalized, pooled, and sequenced on the Illumina Hiseq4000 sequencer (2 × 150 bp paired-end). Libraries were prepared using Library Quant Kit Illumina GA revised primer-SYBR Fast Universal (KAPA, Wilmington, MA, USA) and sequenced for 600 cycles on an Illumina MiSeq using the MiSeq Reagent Kit (Illumina, San Diego, CA, USA). For bioinformatics analysis, high-quality reads were selected, and effective reads were clustered into OTUs based on 97% sequence similarity. Sequence read processing was performed using Quantitative Insights into Microbial Ecology (QIIME) (version 1.9.0). Low-quality reads that did not align to the reference alignment were removed. Chimeras identified using Chimera.UCHIME were also eliminated. Taxonomic assignment of individual datasets was performed using the SILVA128. Operational taxonomic units (OTUs) were generated using Vsearch v1.11.1 with a dissimilarity cutoff of 0.03. Bacterial diversity within the community, such as the relative abundance of bacterial flora, species richness, and evenness, was determined using alpha diversity (*α*-diversity) indices, including Shannon index, observed species, and rank abundance curve with QIIME. To demonstrate variation among different treatment groups, beta-diversity (*β*-diversity) indices were determined, including cluster dendrogram and principal coordinate analysis (PCoA) using QIIME.

### Statistical Analysis

All the statistical analysis was performed with GraphPad Prism 7.04 software (La Jolla, CA, USA). The normal distribution for parametric data is elucidated with the D’Agostino & Pearson normality test. Statistical significance was determined by using analysis of variance (ANOVA) one-way and two-way analysis of variance followed by Tukey’s and Bonferroni’s multiple comparison test, and p < 0.05 was considered to be statistically significant.

## Results

### DIP Intervention Reduced HFD-Induced Obesity-Related Parameters in BALB/C Mice

**Figure 2:**
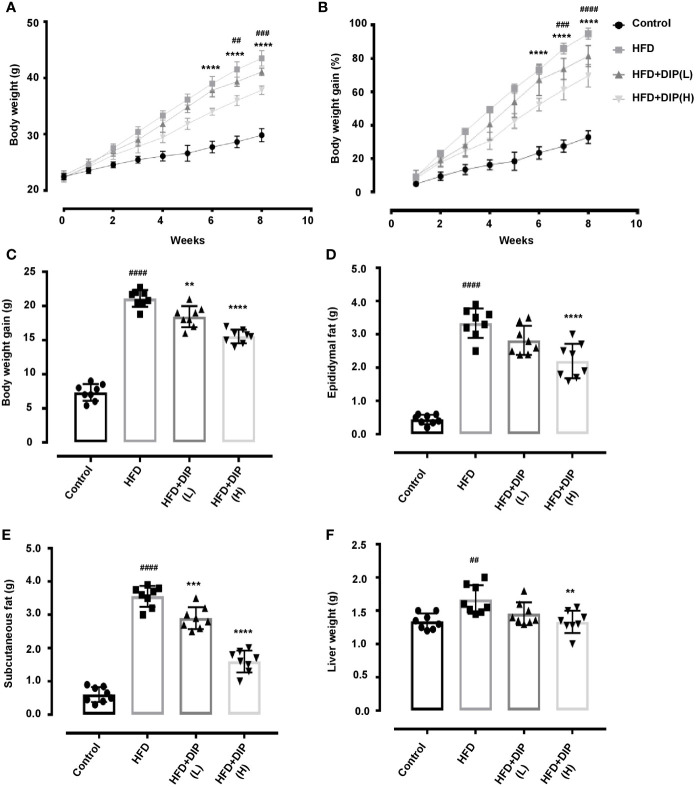
Effect of DIP on body weight and fat accumulation in HFD fed mice. HFD-induced obese mice were treated with 200 µl of DIP at various doses for 4 weeks (n = 8 each group). **(A)** Effects of DIP administration on body weight (g), **(B)** body weight gain (%), **(C)** body weight gain (g), **(D)** epididymal fat (g), **(E)** subcutaneous fat (g), and **(F)** liver weight are presented in the above panels. Data are expressed as mean ± SEM. Body weight differences in **(A, B)** were analyzed using two-way ANOVA Bonferroni *post hoc* test. ^##^p < 0.01, ^###^p < 0.001, ^####^p < 0.0001 HFD group *vs* HFD+DIP(L) group; ****p < 0.0001 HFD group *vs* HFD + DIP(H). One-way ANOVA Bonferroni *post hoc* was employed to determine statistical differences in **(C–F)**. ^##^p < 0.01, ^####^p < 0.0001 HFD group *vs* control group; **p < 0.01, ***p < 0.001, ****p < 0.0001 HFD group *vs* HFD + DIP(L) and HFD + DIP(H) groups.

To study the effect of DIP on obesity-related parameters, we used BALB/C mice model and fed mice with HFD for 8 weeks. HFD feeding for 8 weeks led to a significant increment of the body weight, liver weight, epididymal, and subcutaneous fat accumulation compared with the control group (**Figures 2A–F**). Our findings revealed a significant reduction of body weight, liver weight, epididymal, and subcutaneous fat accumulation in DIP intervention groups in a dose-dependent manner as compared to the HFD alone group (**Figures 2A–F**). The mean body weight was significantly higher in the HFD group (43.5 ± 3 g) as compared to HFD + DIP(L) (41 ± 3 g) and HFD + DIP(H) (38 ± 3 g) groups respectively (**Figure 2A**). The statistically significant weight gain reduction was evidenced after 6 weeks upon DIP supplementation at a high dose (p < 0.0001) and 7 weeks of DIP administration at a low dose (p < 0.01) (p < 0.001) (**Figures 2A, B** **)**. Moreover, epididymal and subcutaneous fat accumulation was significantly reduced in DIP treated groups in a dose-dependent manner (**Figures 2D, E** **)**. A significant increase in liver weight was witnessed in the HFD group (p < 0.01) as compared to the control group. However, a substantial reduction in liver weight was observed at a high dose of DIP (p < 0.01), and HFD + DIP(L) group didn’t reach statistical significance (**Figure 2F**). These findings indicate that DIP decreases weight gain and fat accumulation in HFD-fed mice. Furthermore, food intake and energy intake were assessed in our study (**Supplementary Figure S1**). No significant difference was found among HFD alone and HFD followed by DIP treated groups, hence, suggesting the effect of DIP on obesity-related parameters is not because of less intake of food or energy acquisition. Thus, these findings demonstrate that DIP has the potency to reverse HFD-induced obesity related parameters.

### Histological Examination of Epididymal Adipose Tissue, Determination of Adipogenic and Lipogenic Gene Expression Upon DIP Supplementation

**Figure 3:**
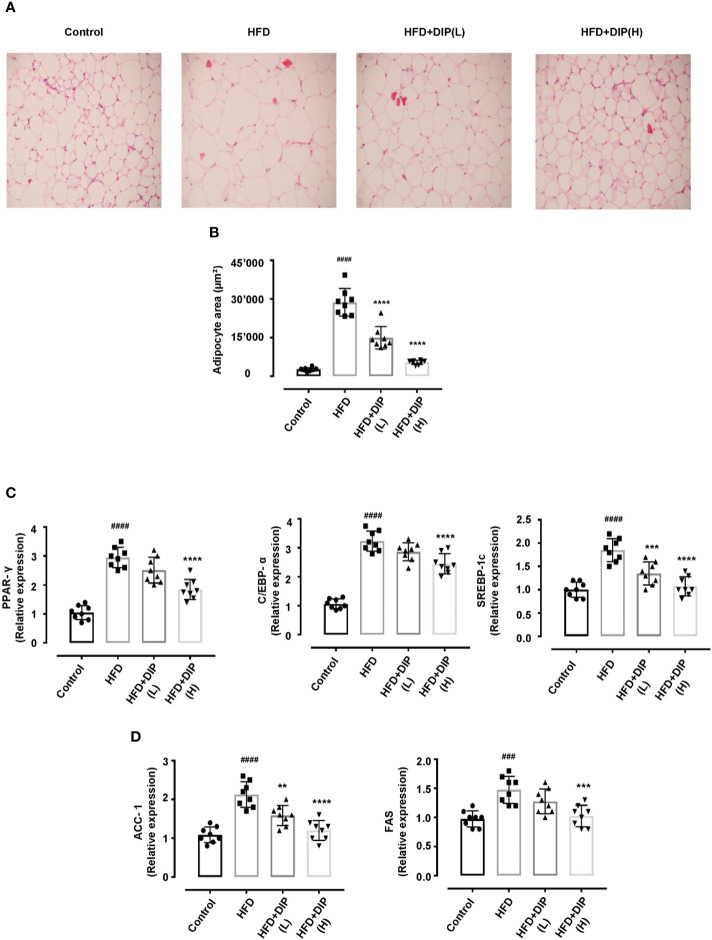
Histologic analysis and lipogenic gene expression of epididymal adipose tissue in HFD-induced obese mice. Adipocyte structural analysis and size estimation through H&E staining and ImageJ software. Scale bar, 50 μm. **(A)** The differences in the histology of adipocytes in various treatment groups are evident in representative H&E stained images. **(B)** The distribution of adipocytes area indicates HFD has greater adipocytes area (>20,000 μm^2^). On the contrary, DIP treated groups revealed smaller adipocytes size (<20,000 μm^2^). Data are expressed as the mean ± SEM, (n = 8) in each group based on one-way ANOVA Tukey *post hoc* analysis. Significant differences were identified at ^####^p < 0.0001, HFD group *vs* control group, and ****p < 0.0001, HFD group *vs* HFD + DIP(L) and HFD + DIP(H) groups. **(C)** Effect of DIP supplementation treatment on the adipogenic genes expression PPAR-*γ*, C/EBP*α*, and SREBP-1c in adipose tissues was determined with qRT-PCR. **(D)** Effect of DIP supplementation treatment on the lipogenic gene expression ACC-1, and FAS in adipose tissues was determined with qRT-PCR. The data is expressed as mean ± SEM (n = 8) based on one-way ANOVA Tukey *post hoc* analysis. Significant differences were identified at ^###^p < 0.001, ^####^p < 0.0001, HFD group *vs* control group; **p < 0.01, ***p < 0.001 and ****p < 0.0001, HFD group *vs* HFD + DIP(L) group or HFD group *vs* HFD + DIP(H) group.

Since body weight gain was reduced after supplementation with DIP in HFD fed mice, we further determined if the bodyweight reduction was due to the decreased fat accumulation. Thus, the weight of epididymal and subcutaneous adipocytes was measured (**Figures 2D, E** **)**, and hematoxylin and eosin (H&E) staining was carried out to examine the histological alteration, *i.e.*, adipocyte size in HFD fed *versus* HFD + DIP treated mice (**Figure 3**). Our finding revealed a significant increase of epididymal and subcutaneous fat accumulation, as presented in **Figures 2D, E**. Moreover, the histological analysis revealed the degree of lipid accumulation in the epididymal adipose tissue, which is proportional to the size of the tissue. The distribution of adipocytes in the HFD group depicted larger adipocyte size as compared to the control group. On the contrary, DIP treatment at a low dose and high dose (p < 0.0001) depicted a significant adipocyte size reduction as compared to the HFD group suggesting the regulatory effect of DIP in fat deposition (**Figure 3B**). These findings were in agreement with previous studies that have reported a higher magnitude of lipid accumulation in HFD fed mice (58, 74).

Multiple research studies have shown that the key regulators of adipogenesis include peroxisome proliferator activated receptor *γ* (PPAR-*γ*), CCAAT/enhancer-binding protein alpha (C/EBP*α*), and sterol response element-binding protein-1c (SREBP-1c) genes (15, 75–78). The overexpression of these genes promotes adipogenesis and induces lipid accumulation. To elucidate the changes at transcriptional level, we determined the expression of these genes in HFD treated mice. In concordance with the earlier findings, our study has shown that expression level of PPAR-*γ*, C/EBP*α*, and SREBP-1c is significantly enhanced in the HFD alone group compared to the control group (**Figure 3C**). However, DIP treatment at low dose (p < 0.001) and at high dose (p < 0.0001) regulated these transcription factors *via* regulation of the adipogenic genes. Furthermore, accumulated studies have suggested that the lipid biosynthesis associated genes including acetyl-CoA carboxylase-1 (ACC-1) and fatty acid synthase (FAS) expression is higher in adipose and liver tissue in HFD fed obese mice (15, 79). Henceforth, we determined the relative expression of these lipogenic genes in the adipose tissue (**Figure 3D**). Our results revealed that the expression level of ACC-1 and FAS significantly increased in the HFD alone group (p < 0.001, p < 0.0001) compared to the control group. Notwithstanding, DIP treatment at low dose (p < 0.01) and at high dose (p < 0.001, p < 0.0001) significantly decreased the expression of lipogenic genes.

### DIP Alleviated Liver Associated Markers and Lipid Profile of HFD-Induced Obesogenic Mice

**Figure 4:**
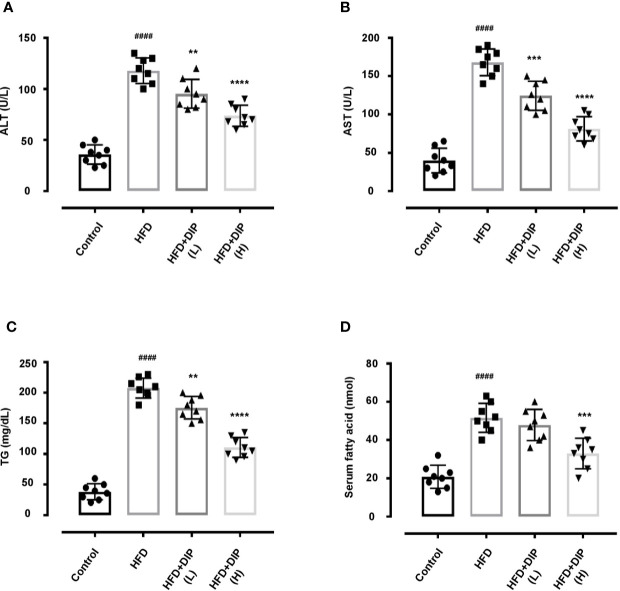
DIP supplementation decreases serum ALT, AST, TG and FFA. Effect of DIP supplementation on serum **(A)** ALT, **(B)** AST, **(C)** TG and **(D)** FFA levels. The data is shown as mean ± SEM (n = 8), based on one-way ANOVA Tukey *post hoc* analysis. Significant differences were identified using at ^####^p < 0.0001 HFD group *vs* control group and **p < 0.01, ***p < 0.001 and ****p < 0.0001, HFD group vs HFD+DIP(L) and HFD+DIP(H) groups.

Previously, studies have shown that the HFD-induced obesity leads to higher serum alanine transaminase (ALT), aspartate transaminase (AST), triglycerides (TGs) and free fatty acid (FFA) levels (15, 80, 81). Therefore, we investigated the effect of DIP intervention on ALT, AST, TG and FFA (**Figures 4A–D**). Our findings revealed that the HFD resulted in significant increase of serum ALT, AST, TG and FFA (p < 0.0001) in the HFD alone group as compared to the control group (**Figure 4**). However, DIP treatment ameliorated liver health by reducing ALT (p < 0.01) DIP + HFD(L), (p < 0.0001) DIP + HFD(H), and AST levels (p < 0.001) DIP + HFD(L), (p < 0.0001) DIP + HFD(H). Moreover, lipid markers such as TG (p < 0.01) DIP + HFD(L), (p < 0.0001) DIP + HFD(H), and FFA (p < 0.001) DIP + HFD(H) were also attenuated significantly. Altogether, our results infer that DIP attenuates liver and lipogenic markers in obesogenic mice.

### DIP Supplementation Ameliorated Serum LPS, Glucose, and Insulin Levels and Improves Intestinal Tight Junction Protein (TJP) Expression in HFD-Induced Obese Mice

**Figure 5:**
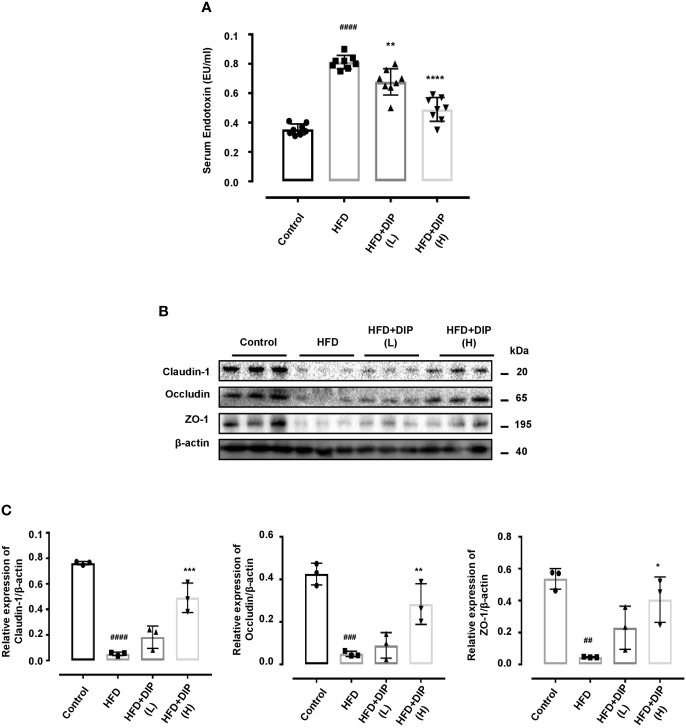
DIP administration reduced serum endotoxin levels and ameliorated intestinal tight junction protein (TJP) expression in HFD fed mice. **(A)** Effect of DIP supplementation on serum LPS levels. Serum endotoxin (EU/ml) was determined using the limulus amebocyte lysate assay kit. The data is shown as mean ± SEM (n = 8), based on one-way ANOVA Tukey *post hoc* analysis. Significant differences were identified at ^###^p < 0.0001 HFD group *vs* control group and **p < 0.01, ****p < 0.0001, HFD group *vs* HFD + DIP(L) and HFD + DIP(H) groups. **(B)** Immunoblots presenting TJP expression in different treatment groups from the colon tissues. **(C)** Bar graph demonstrating relative band intensity of respective protein quantified *via* NIH image J software. The data was acquired from three independent experiments and is expressed as mean ± SEM (n = 8). ^##^p < 0.01, ^###^p < 0.001 ^####^p < 0.0001 HFD group *vs* control group; *p < 0.05, **p < 0.01, ***p < 0.001 HFD group *vs* HFD + DIP(L) or HFD + DIP(H) groups.

Previously, studies have reported that the HFD-induced obesity leads to higher serum lipopolysaccharide (LPS) levels (13, 15). Therefore, we investigated the effect of DIP intervention on serum endotoxin levels (**Figure 5A**). Our findings revealed that HFD resulted in elevated serum endotoxin levels (p < 0.0001) in the HFD group as compared to the control group (**Figure 5A**). However, DIP treatment led to a significant reduction of LPS levels in DIP + HFD(L) (p < 0.01) and DIP + HFD(H) (p < 0.0001) groups. Moreover, glucose and insulin levels were also elevated in obesity (58, 74, 82, 83). To determine DIP outcomes on glucose homeostasis, we assessed fasting glucose and insulin levels. Our findings demonstrated a significant increase in fasting glucose and insulin levels in HFD alone group as compared to the control group (**Supplementary Figure S2**). Nonetheless, upon supplementation with DIP, glucose and insulin levels were reduced markedly.

The fundamental role of tight junction proteins in maintaining mucosal integrity is widely recognized, given that higher endotoxin levels increase the intestinal permeability and disrupt mucosal integrity (15). Hence, we comprehensively analyzed the tight junction protein (TJP) expression including claudin-1, occludin, and zonula occluden (ZO-1) of four experimental groups (**Figures 5B, C**). Immunoblot analysis was performed to demonstrate the robust alterations in TJP expression. The HFD alone group presented decreased TJP expression as compared to the control group (**Figures 5B, C** **)**. However, after DIP intervention, the expression patterns of claudin-1, occludin, and zonula occluden (ZO-1) were upregulated significantly at high dose, and partial modulatory effect was observed at a low dose. Conclusively, our data suggest that HFD-induced obesity resulted in increased mucosal permeability and reduced TJP expression; nonetheless, DIP supplementation had a modulatory effect on intestinal integrity *via* decreasing endotoxin levels and increasing TJP expression.

### DIP Supplementation Reduced Inflammatory Cytokines in Obese Mice

**Figure 6:**
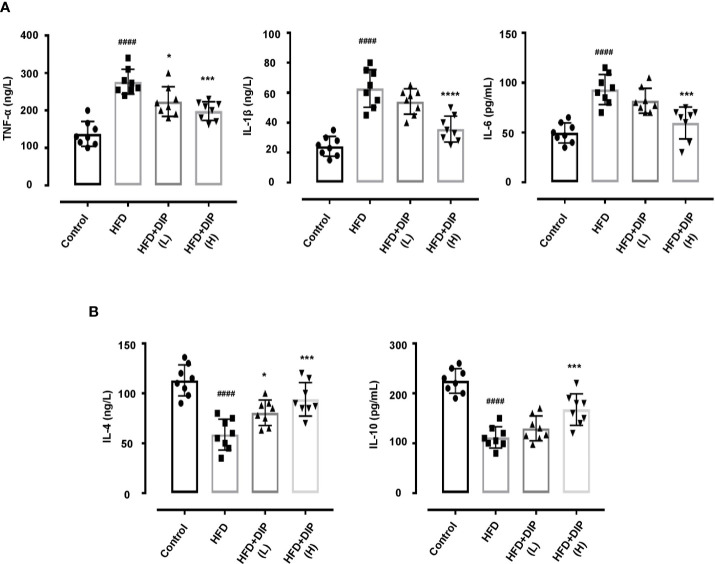
Effect of DIP on pro-inflammatory and anti-inflammatory cytokines in the serum. **(A)** Pro-inflammatory cytokines TNF-α, IL-6, IL-1β. **(B)** Anti-inflammatory cytokines IL-4 and IL-10 levels in the serum were analyzed by ELISA kits. The data was acquired from three independent experiments and is presented as mean ± SEM (n = 8). ^####^p < 0.0001 HFD group *vs* control group; *p < 0.05, ***p < 0.001 and ****p < 0.0001 HFD group *vs* HFD + DIP(L) or HFD + DIP(H) groups.

Previously, *in-vivo* studies have suggested that pro-inflammatory cytokine secretion is elevated in obese animals as compared to non-obese animals (58). Moreover, the upsurge of pro-inflammatory cytokines results in chronic inflammation and insulin resistance (7, 16, 58, 84). Hence we measured the level of pro-inflammatory including tumor necrosis factor-α (TNF-α), interleukin (IL-1β), (IL-6) and anti-inflammatory cytokines (IL-4, IL-10) (**Figure 6**). In concordance with the previous findings, we also found that TNF-α, IL-1β, and IL-6 levels were significantly enhanced in the HFD alone group as compared to the control group. On the contrary, anti-inflammatory cytokine secretion, such as IL-4 and IL-10 levels, was reduced in the HFD group. Nonetheless, DIP treatment significantly reduced the level of pro-inflammatory cytokines and enhanced anti-inflammatory cytokines in DIP supplemented groups in a dose-dependent manner (**Figure 6**). The results revealed that DIP supplementation decreases the inflammatory markers in the obesogenic mice model.

### DIP Administration Reduced Inflammatory Markers in Epididymal Adipose Tissue and Liver of Obese Mice

**Figure 7:**
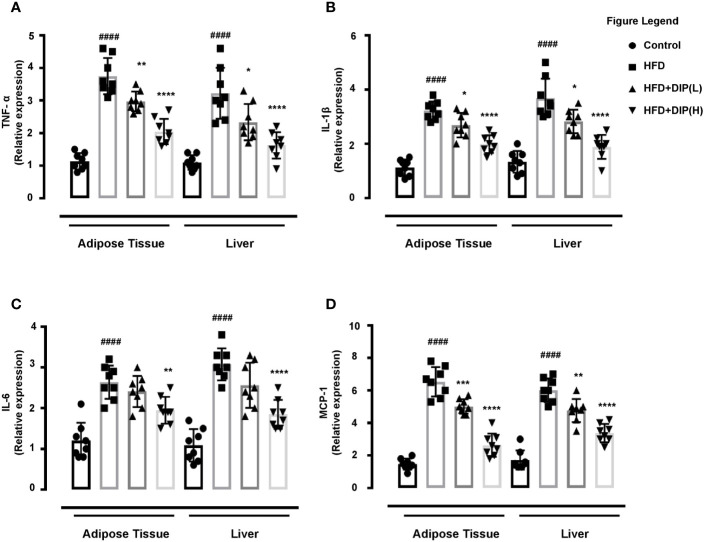
Effect of DIP on adipokine expression in epididymal adipose tissue and liver. The relative mRNA expression of **(A)** TNF-α **(B),** IL-6, **(C)** IL-1β, and **(D)** MCP-1 expression levels in the epididymal adipose tissue and hepatic tissue were analyzed with qPCR. The data is expressed as mean ± SEM (n = 8) based on one-way ANOVA Tukey *post hoc* analysis. Significant differences were identified at ^####^p < 0.0001, HFD group *vs* control group; *p < 0.05, **p < 0.01, ***p < 0.001 and ****p < 0.0001, HFD group *vs* HFD + DIP(L) group or HFD group *vs* HFD + DIP(H) group.

Multiple studies have demonstrated that adipose tissue of an obese animal has shown higher expression of pro-inflammatory cytokines such as TNF-α, IL-1β, and IL-6 levels (16, 58). We assessed the mRNA expression of these inflammatory cytokines in epididymal adipose tissue and liver. In harmony with the previous studies, we found a significantly higher expression level of TNF-α, IL-1β, and IL-6 in the HFD group in comparison with the control group (**Figures 7A–C**). The relative expression of these inflammatory cytokines was notably reduced by DIP treatment in a dose-dependent manner (p < 0.05, p < 0.01, p < 0.0001) (**Figures 7A–C**).

Monocyte chemoattractant protein-1 (MCP-1) is a pro-inflammatory cytokine that has been linked with chronic inflammation through activation of M1 macrophages in obese mice models (85, 86). Henceforth, we determined the mRNA level of MCP-1in the epididymal adipose tissue and liver. In agreement with the earlier studies, we also observed a statistically significant higher mRNA expression of the MCP-1in the HFD group compared to the control group. However, DIP treatment significantly reduced MCP-1 expression levels at low and high doses (p < 0.01, p < 0.001, p < 0.0001) (**Figure 7D**). Thus our findings revealed that DIP supplementation remarkably reduced inflammatory markers and macrophage infiltration in a dose-dependent manner in HFD treated mice.

### DIP Supplementation Restored Gut Microbiota in Obese Mice

**Figure 8:**
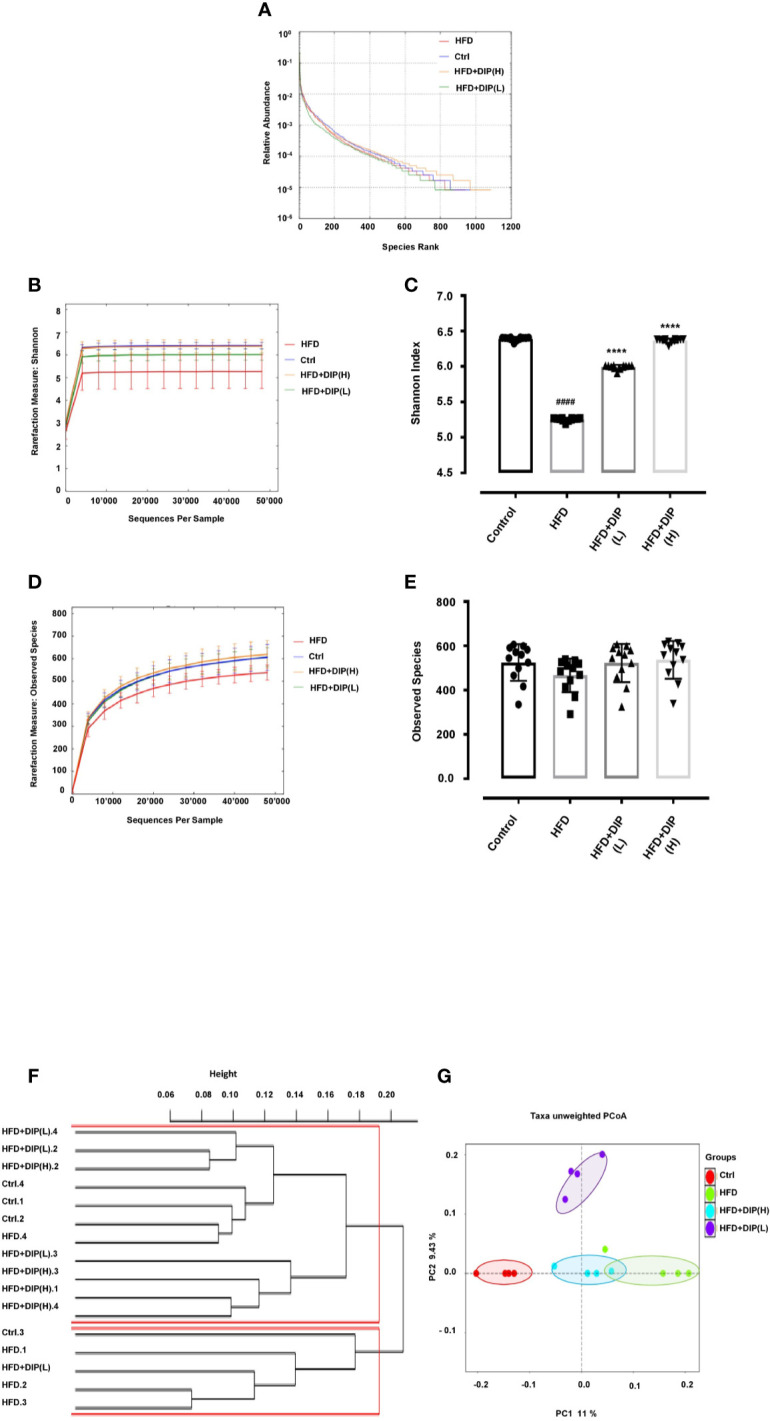
Impact of DIP on gut microbiota diversity and abundance in HFD mice. **(A)** Rank abundance curve, **(B)** Shannon index, **(C)** bar graph for Shannon index, **(D)** observed species, **(E)** bar graph for observed species showing species diversity, richness, and abundance. **(F)** Cluster dendrogram and **(G)** principal coordinate analysis (PCoA) representing the level of similarities or dissimilarities among different treatment groups (n = 4). The data is presented as the mean ± SEM, (n = 4) using one-way ANOVA followed by the Tukey *post hoc* test. Significant differences were identified at ^####^p < 0.0001, HFD group *vs* control group; ****p < 0.0001, HFD group *vs* HFD + DIP(L) group or HFD group *vs* HFD + DIP(H) group. The clustering of PCoA analysis is statistically supported by the one-way ANOVA using ANOSIM test (*R* = 0.4722, *p* = 0.002).

Several reports have shown the intestinal microbiome perturbation (dysbiosis) in obesity, often characterized by reduced bacterial diversity and abundance, along with the alteration of the gut microbiota composition, *i.e*., increased Firmicutes to Bacteroidetes ratio (15, 42, 87). In order to determine the effect of DIP intervention in HFD-induced obese mice, Illumina MiSeq sequencing of 16S r RNA (V3–V5) has been employed in this study. A total of 9,998 operational taxonomic units (OTUs) ranging from 520 to 756 were obtained. The total sequence read count was 1,644,813, and after quality filtering, 1,339,583 clean sequencing reads were acquired. All the samples attained deep coverage (coverage = 1) (**Supplementary Table S2**). The alpha and beta diversity indices have been assessed to demonstrate the bacterial diversity, species richness, and similarities or dissimilarities between different treatment groups. The rank abundance curve depicting species diversity and richness in descending order were as follows: The HFD + DIP(H) group, control group, HFD group, and HFD + DIP(L) group (**Figure 8A**). Meanwhile, rarefaction curves (Shannon index and observed species) show a saturation plateau with different depths for the respective group (**Figures 8B–E**). The higher species richness and abundance were witnessed for the control and HFD + DIP(H) groups followed by the HFD + DIP(L) group. The Shannon index of the HFD group was significantly lower than the control and HFD + DIP(H) group (p < 0.0001) (**Figure 8C**). The HFD group displayed the lowest observed species level as compared to the control and DIP treated groups (**Figures 8D, E** **)**. However, no statistical difference was witnessed in observed species for all the experimental groups. Furthermore, *β*-diversity parameters were analyzed to measure the distance between each sample and the similarities between four experimental groups. The cluster dendrogram and principal coordinate analysis (PCoA) based on unweighted UniFrac distances depicted an evident clustering of bacterial taxa for each treatment group (**Figures 8F, G** **)**. The control and DIP treated groups showed apparent clustering depicting more close association and similarity in comparison with the HFD group. The analysis of similarity (ANOSIM) test using Bray–Curtis dissimilarity revealed that the observed cluster patterns were significant (*R* = 0.4722, *P* = 0.002).

### HFD-Induced Bacterial Taxa Alteration Were Restored Upon DIP Administration

**Figure 9:**
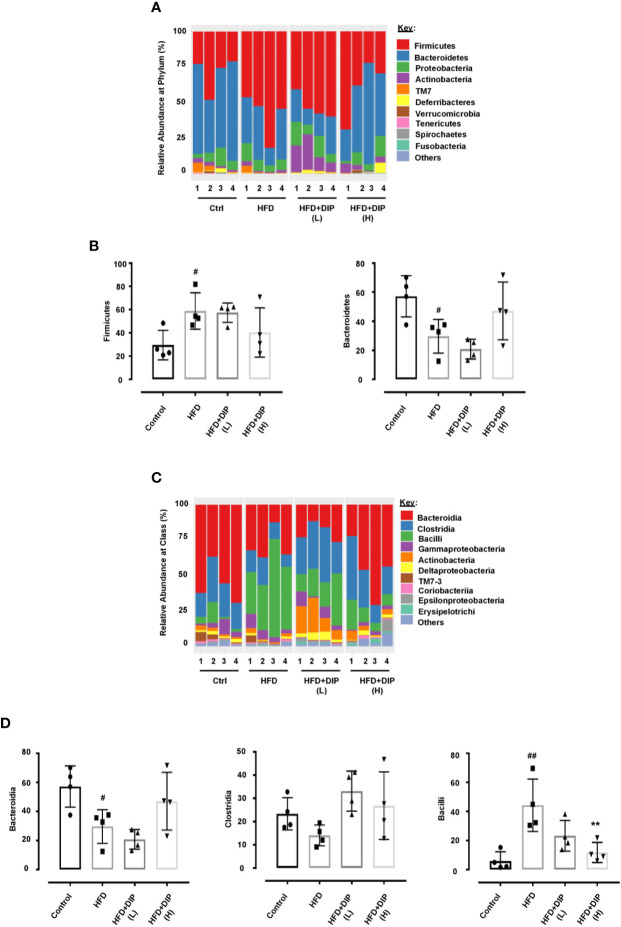
The relative abundance of gut microbiota at phylum and class level in HFD mice. **(A)** The relative abundance of bacterial taxa at the phylum level. **(B)** Abundant flora at the phylum level (%). **(C)** Relative abundance of bacterial taxa at the class level. **(D)** Abundant flora at the class level (%). The data is shown as mean ± SEM, (n = 4) using one-way ANOVA followed by the Tukey *post hoc* test. Significant differences were identified at ^#^p < 0.05, ^##^p < 0.01, HFD group *vs* control group, and **p < 0.01, HFD group *vs* HFD + DIP(H) group.

At the phylum level, a total of ten bacterial phyla were detected, and among them, there were five dominated phyla: Firmicutes, Bacteroidetes, Proteobacteria, Actinobacteria, and TM7 (**Figure 9A**) (**Supplementary Table S5**). Previous studies have shown an increment of Firmicutes over Bacteroidetes in obese patients and HFD-induced obese mice (42, 87–89). In concordance with these studies, we also observed a relative increase abundance of Firmicutes to Bacteroidetes ratio in the HFD alone group as compared to the control group (**Figure 9A**). The higher abundance of Firmicutes and decreased abundance of Bacteroidetes were statistically significant in the HFD group in comparison with the control group (p < 0.05) (**Figure 9B**). It is noteworthy that the bacterial taxonomy at the phylum level was reversed after DIP treatment, particularly at a high dose. The level of Firmicutes decreased, and Bacteroidetes increased after DIP treatment partially at a low dose and markedly at a high dose showing a similar trend like the control subjects. However, statistical significance was not achieved at the given doses of DIP.

Moreover, dynamic alterations were observed at the class level, where the major phyla were *Bacteroidia, Clostridia, Bacilli, Gammaproteobacteria, and Actinobacteria* (**Figure 9C**) (**Supplementary Table S6**). The HFD alone group presented a higher abundance of *Bacilli* (belongs to Firmicutes phylum) and *Gammaproteobacteria* (belongs to Proteobacteria phylum) and lower levels of *Bacteroidia* (belongs to Bacteroidetes phylum). The lower level of *Bacteroidia* (p < 0.05) and increment of *Bacilli* (p < 0.01) were statistically significant in the HFD group as compared to the control group (**Figure 9D**). However, DIP treatment led to the restoration of altered microbiome composition, particularly at a high dose, which presented similar bacterial composition to that of the control group that were fed a standard chow diet. Overall, our findings revealed that HFD led to gut microbiota dysbiosis. Nonetheless, given DIP treatment, the dynamic bacterial perturbations were restored.

## Discussion

Obesity is characterized by systemic inflammation and is associated with the etiology of multiple chronic illnesses such as diabetes, cardiovascular diseases, liver inflammation, metabolic disorders, and various tumors (6, 7, 14, 90–92). The prevalence of obesity is increasing immensely around the globe. Henceforth, researchers are trying to develop novel anti-obesity arsenals to combat and halt this rate. Multiple studies have reported the therapeutic potential and health-promoting properties of polysaccharides from natural dietary sources such as mushrooms, seaweed, cereals, and herbs (93–99). The role of polysaccharide from edible and medicinal mushrooms in treating various illnesses is well documented (15, 58, 96). Among them, *D. indusiata* is an edible, as well as a medicinal mushroom that possesses several biological properties (64, 67, 100–102). To date, limited research has been done on the biological effect of *D. indusiata* in reducing obesity. Therefore we have used HFD-induced obesity mouse model to unravel the effect of *D. indusiata* polysaccharide (DIP) in reducing the dietary-induced obesity *via* modulating intestinal microbiota and inflammatory-related signaling pathways. In this study, our findings demonstrated that DIP polysaccharide reduced HFD-induced obesity parameters and inflammatory cascades *via* restoring intestinal microbial shift, regulating inflammatory reactions, and modulating intestinal integrity.

Excessive lipid accumulation is one of the major factors in the development of obesity (103). In our study, HFD feeding led to a significant increment of body weight, epididymal and subcutaneous fat (**Figure 2**). Moreover, a substantial increase in liver weight was observed in the HFD alone group in comparison with the control group. However, DIP treatment reduced body weight gain significantly on the 7^th^ and 8^th^ weeks. Furthermore, fat deposition and liver weight were also reduced significantly at a high dose of DIP. These results indicate the protective effect of *D. indusiata* derived polysaccharide in reducing HFD-induced obesity parameters. Research literature shows that HFD-induced obesity often results in hyperinsulinemia and hyperglycemia (15, 104). As expected, our study showed elevated fasting glucose and insulin levels (**Supplementary Figure S2**). Interestingly, DIP supplementation at various doses attenuated the higher levels of glucose and insulin in HFD mice.

Several studies have suggested the enlarged epididymal adipose tissue size and lower number of adipocytes in obese individuals and in *in-vivo studies* (15, 105–107). Moreover, changes in the cascades of transcriptional factors, such as higher expression of adipogenic (PPAR-*γ*, C/EBP*α*, SREBP-1c) and lipogenic genes (ACC-1, FAS) are documented in obesity associated studies (15, 75–79). In corroboration with these studies, we also found an increment of adipocytes size and increase expression of adipogenic and lipogenic genes in the HFD alone group in comparison with the control group. On the contrary, DIP treatment resulted in reducing adipocyte area and decreasing the expression of adipogenic genes in a dose-dependent manner (**Figure 3**). Additionally, hypertrophic adipocytes are attributed to secrete pro-inflammatory cytokines during the development of obesity (108, 109). The increased production of pro-inflammatory cytokines such as “TNF- α, IL-1β, IL-6, and MCP-1” in turn, triggers chronic inflammatory reactions and enhance insulin resistance (7, 84). Thus, regulation of inflammatory cytokine production is one of the most critical factors to reduce inflammatory reactions and insulin resistance. In the present study, we observed a higher production of pro-inflammatory cytokines (TNF-α, IL-1β, IL-6, and MCP-1) (**Figures 6** and **7**) and lower levels of anti-inflammatory cytokines (IL-4, IL-10) (**Figure 6**) upon HFD feeding. Interestingly, DIP intervention for four weeks antagonized the inflammatory reactions by reducing pro-inflammatory cytokines and increasing anti-inflammatory cytokines.

HFD feeding induces liver injury by elevating liver associated biomarkers such as (ALT and AST) and also enhances TG and FFA due to increased adiposity (15, 80, 81, 110). In agreement with the earlier findings, in our study, we also found higher levels of ALT, AST, TG, and FFA in HFD mice (**Figure 4**). Nonetheless, we observed that DIP reduced these markers in a dose-dependent manner suggesting the efficacy of DIP polysaccharide in ameliorating liver health and adiposity.

The higher level of pro-inflammatory cytokines as a result of high-fat diet intake are well known to trigger inflammatory reactions that tend to disrupt intestinal barrier, reduced TJP expression, and increased endotoxemia (LPS). The HFD induced increased inflammatory cytokine production, and increased level of LPS levels in blood circulation leads to systemic and low grade inflammation *via* activation of the TLR4 signaling pathway (46). Our study demonstrated similar outcomes in HFD-induced obese mice. The endotoxin levels were quite enhanced in the HFD alone group comparatively to the control and DIP treated groups (**Figure 5A**). Moreover, TJP expression was down-regulated in the HFD alone group (**Figures 5B, C** **)**. On the contrary, DIP supplemented groups have shown decreased LPS levels and upregulated expression of TJPs compared to the HFD alone group. Altogether, these results suggested the modulatory effect of DIP supplementation in ameliorating intestinal integrity and inflammatory markers.

The intestinal microbiome is receiving much attention in recent years as studies have shown the association of gut microbiota in several inflammatory and obesity-related disorders (111–114). One of the most significant factors in obesity is the composition of the intestinal microbiome as gut microbes play an essential role in nutrient acquisition, vitamin production, energy, and fat storage regulation (27, 28, 115). Therefore, to determine the link between gut microbiota and obesity may provide a much deeper insight into dealing with obesity-related health issues. Diet has a significant impact on the composition of the intestinal microbiome. HFD has been reported to cause an imbalance of intestinal microbiome composition in obese individuals, and the disruption of intestinal microbiome homeostasis has been linked to the progression of obesity and related metabolic disorders (38, 116). On the contrary, multiple studies have shown the modulatory effect of fiber-rich diet such as prebiotics, polyphenols, oligosaccharides, and polysaccharides on gut microbiome community (24, 48, 49, 58, 117). Manipulation of microbial flora through dietary fiber (prebiotics) and non-digestible oligosaccharides or polysaccharide can positively influence the microbial community by promoting the growth of beneficial flora (118, 119). To demonstrate the impact of polysaccharide treatment on gut microbiome community in HFD mice, we employed 16S rRNA Illumina miSeq sequencing. Our results showed that polysaccharide supplementation positively changed the signature of the intestinal microbiome in DIP intervention groups. On the contrary, we found reduced OTUs at all taxonomic levels in HFD alone group compared to the control and DIP treated groups (**Supplementary Table S4**). The alpha and beta diversities were assessed to determine the bacterial diversity, richness, abundance, and similarities among various treatment groups (**Figure 8**) (**Supplementary Table S3**). Previous studies have suggested lower bacterial diversity and richness in obese individuals in comparison with lean individuals (41, 120). The alpha diversity indices were measured with the rank abundance curve, Shannon index, and observed species. In agreement with the earlier findings, our study reflected similar outcomes, *i.e.*, the HFD alone group presented the lowest bacterial diversity followed by HFD + DIP(L). The highest bacterial diversity was observed for the control and HFD + DIP(H) group suggesting the beneficial effect of polysaccharide intervention. Moreover, the distribution pattern of microbial communities in various treatment groups presented as beta diversity indices (cluster dendrogram and PCoA) (**Figures 8F, G** **)**. The cluster dendrogram and PCoA analysis revealed an obvious clustering of all the experimental groups, and the difference was statistically significant. The microbial composition in the control group and DIP intervention groups showed more closeness compared to the HFD alone group. HFD group clustered far from the control and DIP supplemented groups indicating the significance of polysaccharide ingestion in the diet as a modulatory compound for the bacterial community. Approximately 90% of the gut microbiota is composed of Firmicutes, Bacteroidetes, Proteobacteria, and Actinobacteria. Previous studies have shown alteration in the composition of these most abundant gut flora, *i.e.*, higher Firmicutes to Bacteroidetes ratio in obese mice models (42, 87). In our study, Firmicutes, Bacteroidetes, Proteobacteria, Actinobacteria, TM7, and Deferribacteres were evidenced where Firmicutes and Bacteroidetes were the most abundant flora at Phylum level. At the class level, the major phyla were *Bacteroidia, Clostridia, Bacilli*, and *Gammaproteobacteria*. Overall, perturbation of bacterial flora was observed at Phylum and Class levels in the HFD group. In agreement with previous studies, we also found an increased abundance of Firmicutes and reduced Bacteroidetes levels in the HFD alone group (**Figures 9A, B** **)**. Moreover, at the class level, the HFD group depicted an increased abundance of *Bacilli, Gammaproteobacteria* (belongs to Firmicutes and Proteobacteria) and decrease abundance of *Bacteroidia* (belongs to Bacteroidetes) compared to the control and DIP treated groups (**Figures 9C, D** **)**. Interestingly HFD-induced bacterial alterations were reversed by DIP supplementation partially at low dose and greatly at a high dose, suggesting the modulatory impact of polysaccharide supplementation on gut microbiome composition.

In our research, we adopted the approach used by Liang Z et al., Lee H-G et al., and An Y et al. (71, 121, 122) and therefore, we haven’t randomized the treatment within each cage and kept each individual cage for one specific treatment *i.e.*, one cage for the high dose treatment and another for the low dose treatment. However, randomizing the treatment within the cages could be another efficient approach, which can help to eliminate the cage/litter effect as well. Hence, we will adopt this methodology in future studies and will compare the outcomes with the reported results.

## Conclusions

In conclusion, our study demonstrated that HFD supplementation for eight weeks led to obesity in mice that is associated with increased body weight, epididymal and subcutaneous fat accumulation, increase fasting glucose and insulin levels, elevated lipogenic gene expression, increase inflammatory reactions, such as high endotoxin (LPS) and pro-inflammatory cytokines secretion, reduced TJP expression and gut dysbiosis. *D. indusiata* derived crude polysaccharide (DIP) supplementation resulted in alleviating HFD-induced obesity by mitigating obesity related parameters. The inflammatory cascades, such as the increment of pro-inflammatory cytokines, elevated lipogenic genes expression and higher endotoxin levels, were regulated. The expression levels of TJPs were enhanced, and the inflammatory reactions were modulated. The bacterial taxa shift is corrected by reducing the abundance of bacterial taxa involved in obesity, such as Firmicutes, Clostridia, and Bacilli. Comprehensively, our findings suggested that *D. indusiata* possesses anti-obesogenic and anti-inflammatory properties. However, further research is needed on the purification and identification of the bioactive compounds of crude polysaccharide from *D. indusiata* that are responsible for mitigating obesity and associated parameters in a mice model as well as to test the effect of DIP in clinical trials.

## Data Availability Statement

The datasets used and analyzed during the current study are available from the corresponding author on reasonable request.

## Ethics Statement

The animal study was reviewed and approved by Dalian Medical University Committee for animal experiments.

## Author Contributions

SK and YX conceived the study. YX attained funds and supervised the project. SK and SA designed the experiments. SK performed the experiments. SK acquired and analyzed the data. SK wrote the original article, which was revised critically by SA and YX. All authors contributed to the article and approved the submitted version.

## Funding

This research was funded by the Chinese Scholarship Council grant number CSC no: 2016DFH376, and “The APC was supported by Liaoning Provincial Program for Top Discipline of Basic Medical Sciences”.

## Conflict of Interest

The authors declare that the research was conducted in the absence of any commercial or financial relationships that could be construed as a potential conflict of interest.
