# Supplementary material for: Anti-Obesity Effect of Dictyophora indusiata Mushroom Polysaccharide (DIP) in High Fat Diet-Induced Obesity via Regulating Inflammatory Cascades and Intestinal Microbiome
Source: Front Endocrinol (Lausanne). 2020 Nov 19;11:558874. doi: 10.3389/fendo.2020.558874 (PMC7717937; doi:10.3389/fendo.2020.558874)
Supplement: Supplementary file 1 [file DataSheet_1.pdf]

Supplementary Material

1.1 Supplementary Figures

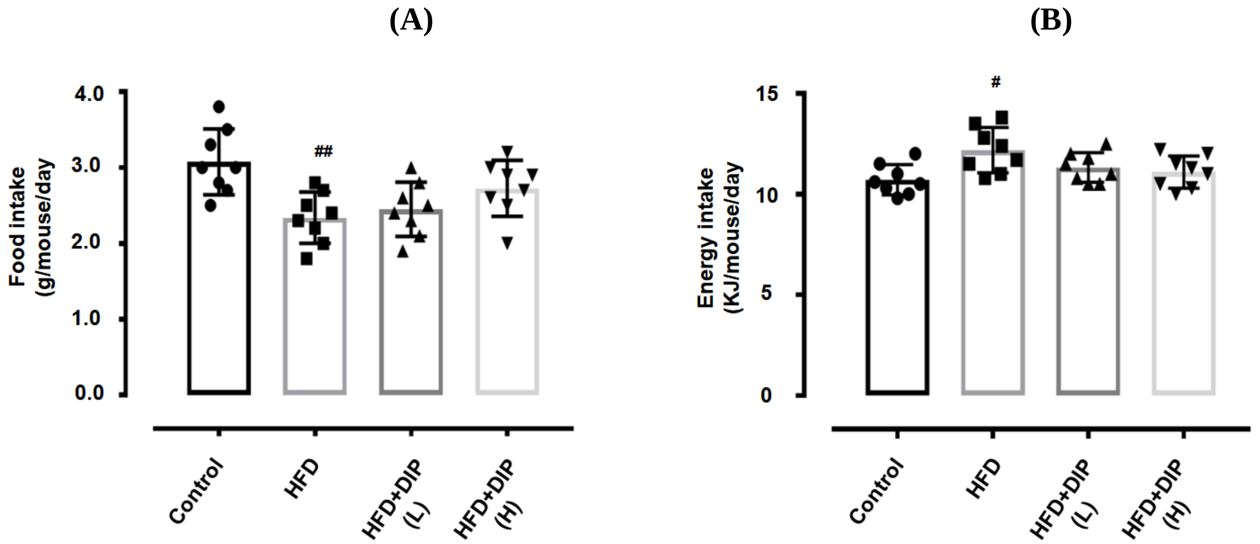

Supplementary Figure S1. Effects of DIP on food intake was monitored in HFD-fed mice. (A) Food intake and (B) Energy intake in different treatment groups (n=8 each group). Statistical significance was assessed by one-way analysis of variance (ANOVA) followed by Tukey's multiple comparison test and is represented as follows: # p < 0.05, ## p < 0.01 HFD group vs. control group.

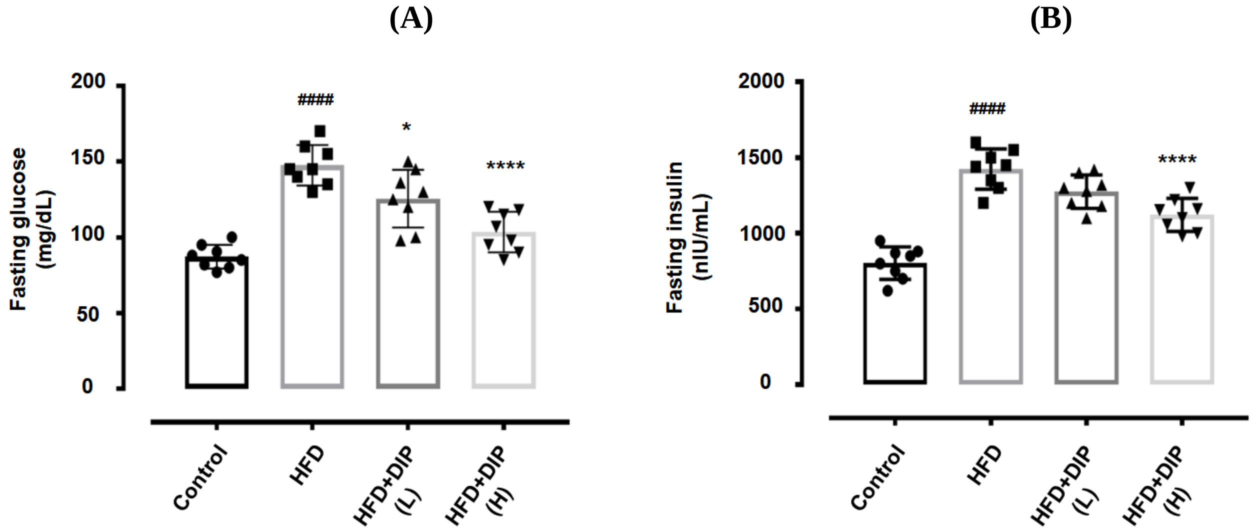

Supplementary Figure S2: Protective Effect of DIP on fasting glucose and insulin levels in HFD induced obese mice. (A) Fasting glucose, and **(B)** Fasting insulin levels were determined using glucose meter and commercial ELISA kit. The data are presented as the means ± standard error of mean SEM (n = 8). Statistical significance was assessed by one-way analysis of variance (ANOVA) followed by Tukey's multiple comparison test and is represented as follows: #### p < 0.0001 HFD group vs. control group; * p < 0.05, **** p < 0.0001 HFD group vs HFD+DIP(L) or HFD+DIP(H) groups.

1.2 Supplementary Tables

Supplementary Table S1: qRT-PCR primer sequences

Name	Sequence
**TNF-α Forward**	5'-TAGCCAGGAGGGAGAACAGA-3'
**TNF-α Reverse**	5'-TTTTCTGGAGGGAGATGTGG-3'
**IL-1β Forward**	5'-TTGAAGAAGAGCCCATCCTC -3'
**IL-1β Reverse**	5'-CAGCTCATATGGGTCCGAC -3'
**IL-6 Forward**	5'-CCGGAGAGGAGACTTCAC-3'
**IL-6 Reverse**	5'-TCCACGATTTCCCAGAGA-3'
**MCP-1 Forward**	5'-TCACTGAAGCCAGCTCTCTCT -3'
**MCP-1 Reverse**	5'-GTGGGGCGTTAACTGCAT-3'
**PPAR-γ Forward**	5'-GCAGCTACTGCATGTGATCAAGA-3'
**PPAR-γ Reverse**	5'-GTCAGCGGGTGGGACTTTC-3'
**C/EBPα Forward**	5'- CGCAAGAGCCGAGATAAAGC-3'
**C/EBPα Reverse**	5'- CACGGCTCAGCTGTTCCA-3'
**SREBP-1c Forward**	5'-GATGTGCGAACTGGACACAG-3'
**SREBP-1c Reverse**	5'-CATAGGGGGCGTCAAACAG-3'
**ACC-1 Forward**	5'-GAGTGACTGCCGAAACATCTCTG-3'
**ACC-1 Reverse**	5'- GCAAGGAGGACAGAGTTTATCGTG-3'
**FAS Forward**	5'-GCTGCGGAAACTTCAGGAAAT-3'
**FAS Reverse**	5'-AGAGACGTGTCACTCCTGGACTT-3'

Supplementary Table S2: OTUs sequencing summary attained in the study

Groups	Sample Size	Singleton %	Chimeras %	Clean tags	OTU,s	Coverage
**Control.1**	98769	82.36	0.18	81313	637	1
**Control.2**	105425	83.85	0.28	86249	573	1
**Control.3**	104480	77.20	0.20	88935	669	1
**Control.4**	101246	77.80	0.40	84518	690	1
**HFD.1**	106129	80.17	0.55	84101	609	1
**HFD.2**	102055	84.01	0.26	81602	568	1
**HFD.3**	102656	77.35	0.20	88415	520	1
**HFD.4**	99299	78.34	0.33	79712	575	1
**HFD+DIP(L).1**	105698	80.82	0.18	87102	536	1
**HFD+DIP(L).2**	100498	74.63	0.28	84514	690	1
**HFD+DIP(L).3**	109045	75.13	0.44	87037	650	1
**HFD+DIP(L).4**	104114	77.93	0.36	85528	685	1
**HFD+DIP(H).1**	98955	77.38	0.37	77690	651	1
**HFD+DIP(H).2**	103489	83.22	0.27	83185	598	1
**HFD+DIP(H).3**	104109	83.66	0.26	80345	591	1
**HFD+DIP(H).4**	98846	79.83	0.23	79337	756	1

Supplementary Table S3: Summary of alpha diversity indices

Groups	Shannon	Simpson	Chao	ACE	Goods coverage
**Control.1**	5.99	0.92	696.80	684.00	1.00
**Control.2**	5.99	0.94	568.57	564.62	1.00
**Control.3**	6.09	0.95	638.75	641.86	1.00
**Control.4**	6.49	0.97	688.50	681.47	1.00
**HFD.1**	6.25	0.97	572.14	572.47	1.00
**HFD.2**	6.26	0.97	517.76	521.13	1.00
**HFD.3**	5.63	0.93	574.83	587.17	1.00
**HFD.4**	6.84	0.98	742.19	744.56	1.00
**HFD+DIP(L).1**	6.29	0.97	578.71	575.21	1.00
**HFD+DIP(L).2**	5.41	0.91	573.56	582.40	1.00
**HFD+DIP(L).3**	5.98	0.95	652.76	640.96	1.00
**HFD+DIP(L).4**	4.01	0.79	504.10	500.54	1.00
**HFD+DIP(H).1**	6.26	0.95	667.84	662.58	1.00
**HFD+DIP(H).2**	6.60	0.98	649.10	637.87	1.00
**HFD+DIP(H).3**	5.66	0.90	656.58	670.77	1.00
**HFD+DIP(H).4**	6.38	0.97	550.79	555.29	1.00

Supplementary Table S4: Number of OTUs at phylum, Class, Order, Family, Genus and Species level

Groups	PHYLUM	CLASS	ORDER	FAMILY	GENUS	SPECIES
**Control.1**	563	563	563	435	208	35
**Control.2**	498	498	498	401	194	33
**Control.3**	575	575	575	453	202	30
**Control.4**	598	598	598	476	199	34
**HFD.1**	537	537	537	400	189	32
**HFD.2**	515	515	515	397	211	34
**HFD.3**	444	444	444	338	178	28
**HFD.4**	510	510	510	420	202	32
**HFD+DIP(L).1**	473	473	473	413	231	41
**HFD+DIP(L).2**	604	604	604	443	236	54
**HFD+DIP(L).3**	570	570	570	462	247	51
**HFD+DIP(L).4**	583	583	583	446	202	35
**HFD+DIP(H).1**	577	577	577	436	211	48
**HFD+DIP(H).2**	514	514	514	432	204	36
**HFD+DIP(H).3**	525	525	525	466	193	30
**HFD+DIP(H).4**	661	661	661	524	244	52

Supplementary Table S5: Bacterial taxa at phylum level (%)

Bacterial flora at phylum level (%)																
	CTRL				HFD				HFD+DIP(L)				HFD+DIP(H)			
	CTRL.1	CTRL.2	CTRL.3	CTRL.4	HFD.1	HFD.2	HFD.3	HFD.4	HFD+DIP(L).1	HFD+DIP(L).2	HFD+DIP(L).3	HFD+DIP(L).4	HFD+DIP(H).1	HFD+DIP(H).2	HFD+DIP(H).3	HFD+DIP(H).4
Taxonomy	%	%	%	%	%	%	%	%	%	%	%	%	%	%	%	%
Unassigned;Other	0.10%	0.00%	0.10%	0.20%	0.10%	0.10%	0.00%	0.10%	0.10%	0.00%	0.00%	0.00%	0.00%	0.10%	0.40%	0.50%
k__Bacteria;p__Actinobacteria	2.60%	2.10%	1.00%	1.10%	2.30%	0.90%	0.60%	2.00%	10.70%	14.30%	5.30%	3.80%	3.90%	2.90%	0.80%	2.60%
k__Bacteria;p__Bacteroidetes	63.90%	37.50%	57.00%	70.10%	32.80%	37.60%	12.50%	35.70%	25.40%	13.20%	16.90%	27.50%	23.00%	47.20%	71.60%	46.40%
k__Bacteria;p__Cyanobacteria	0.10%	0.00%	0.00%	0.00%	0.00%	0.10%	0.00%	0.00%	0.00%	0.00%	0.00%	0.00%	0.00%	0.00%	0.00%	0.00%
k__Bacteria;p__Deferribacteres	0.20%	0.20%	1.30%	0.20%	0.20%	0.10%	0.10%	0.00%	0.30%	1.20%	0.90%	0.30%	0.00%	0.00%	0.20%	3.90%
k__Bacteria;p__Firmicutes	22.90%	48.30%	26.00%	20.90%	46.60%	52.50%	81.80%	54.40%	44.90%	62.40%	60.90%	61.50%	70.70%	38.20%	22.10%	30.70%
k__Bacteria;p__Fusobacteria	0.00%	0.00%	0.00%	0.10%	0.10%	0.10%	0.00%	0.00%	0.00%	0.00%	0.00%	0.00%	0.00%	0.40%	0.00%	0.20%
k__Bacteria;p__Proteobacteria	2.90%	6.60%	13.30%	6.60%	12.70%	8.00%	4.80%	7.40%	18.30%	7.90%	15.70%	6.00%	1.90%	9.00%	4.40%	15.20%
k__Bacteria;p__Spirochaetes	0.00%	0.00%	0.40%	0.00%	0.00%	0.00%	0.00%	0.00%	0.00%	0.00%	0.00%	0.00%	0.00%	0.00%	0.40%	0.20%
k__Bacteria;p__Synergistetes	0.00%	0.00%	0.00%	0.00%	0.00%	0.00%	0.00%	0.00%	0.00%	0.00%	0.00%	0.00%	0.00%	0.10%	0.10%	0.00%
k__Bacteria;p__TM7	6.60%	3.40%	0.40%	0.70%	5.00%	0.60%	0.10%	0.30%	0.20%	0.60%	0.20%	0.70%	0.30%	0.20%	0.00%	0.30%
k__Bacteria;p__Tenericutes	0.60%	0.40%	0.30%	0.10%	0.30%	0.10%	0.20%	0.00%	0.00%	0.30%	0.10%	0.20%	0.10%	0.00%	0.00%	0.00%
k__Bacteria;p__Verrucomicrobia	0.00%	1.50%	0.00%	0.00%	0.00%	0.00%	0.00%	0.00%	0.00%	0.00%	0.00%	0.00%	0.00%	1.80%	0.00%	0.00%

Supplementary Table S6: Bacterial taxa at class level (%)

Bacterial flora at class level (%)																
	CTRL				HFD				HFD+DIP(L)				HFD+DIP(H)			
	CTRL.1	CTRL.2	CTRL.3	CTRL.4	HFD.1	HFD.2	HFD.3	HFD.4	HFD+DIP(L).1	HFD+DIP(L).2	HFD+DIP(L).3	HFD+DIP(L).4	HFD+DIP(H).1	HFD+DIP(H).2	HFD+DIP(H).3	HFD+DIP(H).4
Taxonomy	%	%	%	%	%	%	%	%	%	%	%	%	%	%	%	%
Unassigned;Other;Other	0.10%	0.00%	0.10%	0.20%	0.10%	0.10%	0.00%	0.10%	0.10%	0.00%	0.00%	0.00%	0.00%	0.10%	0.40%	0.50%
k__Bacteria;p__Actinobacteria;c__Actinobacteria	0.80%	0.40%	0.20%	0.10%	1.00%	0.00%	0.20%	0.20%	10.40%	14.10%	5.00%	3.10%	3.10%	0.50%	0.10%	1.50%
k__Bacteria;p__Actinobacteria;c__Coriobacteriia	1.80%	1.70%	0.80%	1.00%	1.30%	0.90%	0.40%	1.80%	0.40%	0.20%	0.30%	0.80%	0.80%	2.40%	0.70%	1.10%
k__Bacteria;p__Bacteroidetes;c__Bacteroidia	63.90%	37.50%	57.00%	70.10%	32.80%	37.60%	12.50%	35.70%	25.40%	13.20%	16.90%	27.50%	23.00%	47.20%	71.60%	46.40%
k__Bacteria;p__Bacteroidetes;c__Flavobacteriia	0.00%	0.00%	0.00%	0.00%	0.00%	0.00%	0.00%	0.00%	0.00%	0.00%	0.00%	0.00%	0.00%	0.00%	0.00%	0.00%
k__Bacteria;p__Bacteroidetes;c__Sphingobacteriia	0.00%	0.00%	0.00%	0.00%	0.00%	0.00%	0.00%	0.00%	0.00%	0.00%	0.00%	0.00%	0.00%	0.00%	0.00%	0.00%
k__Bacteria;p__Cyanobacteria;c__4C0d-2	0.00%	0.00%	0.00%	0.00%	0.00%	0.10%	0.00%	0.00%	0.00%	0.00%	0.00%	0.00%	0.00%	0.00%	0.00%	0.00%
k__Bacteria;p__Cyanobacteria;c__Chloroplast	0.10%	0.00%	0.00%	0.00%	0.00%	0.00%	0.00%	0.00%	0.00%	0.00%	0.00%	0.00%	0.00%	0.00%	0.00%	0.00%
k__Bacteria;p__Deferribacteres;c__Deferribacteres	0.20%	0.20%	1.30%	0.20%	0.10%	0.10%	0.10%	0.00%	0.30%	1.20%	0.90%	0.30%	0.00%	0.00%	0.20%	3.90%
k__Bacteria;p__Firmicutes;c__Bacilli	4.90%	15.20%	1.50%	2.10%	30.40%	32.30%	69.60%	44.90%	13.80%	22.60%	18.50%	38.20%	21.80%	10.40%	6.00%	8.90%
k__Bacteria;p__Firmicutes;c__Clostridia	17.60%	32.80%	24.30%	18.70%	15.70%	19.40%	12.00%	9.10%	28.90%	39.20%	41.30%	23.10%	46.70%	27.60%	12.70%	20.40%
k__Bacteria;p__Firmicutes;c__Erysipelotrichi	0.40%	0.30%	0.20%	0.10%	0.40%	0.70%	0.20%	0.40%	2.20%	0.60%	1.10%	0.20%	2.20%	0.20%	3.40%	1.40%
k__Bacteria;p__Fusobacteria;c__Fusobacteriia	0.00%	0.00%	0.00%	0.10%	0.10%	0.10%	0.00%	0.00%	0.00%	0.00%	0.00%	0.00%	0.00%	0.40%	0.00%	0.20%
k__Bacteria;p__Proteobacteria;c__Alphaproteobacteria	0.00%	0.00%	0.10%	0.00%	0.00%	0.00%	0.00%	0.00%	0.70%	0.00%	0.10%	0.00%	0.20%	0.00%	0.70%	0.30%
k__Bacteria;p__Proteobacteria;c__Betaproteobacteria	0.10%	0.40%	0.10%	0.10%	0.30%	0.40%	0.00%	2.70%	0.10%	0.20%	0.20%	0.20%	0.20%	2.80%	0.10%	1.00%
k__Bacteria;p__Proteobacteria;c__Deltaproteobacteria	1.50%	2.70%	1.20%	2.30%	1.70%	0.50%	0.30%	1.50%	3.00%	6.20%	6.40%	2.10%	0.50%	3.30%	0.80%	2.40%
k__Bacteria;p__Proteobacteria;c__Epsilonproteobacteria	0.20%	0.30%	0.50%	0.30%	0.20%	0.50%	0.70%	0.20%	2.70%	0.60%	0.60%	0.20%	0.00%	0.20%	0.20%	8.80%
k__Bacteria;p__Proteobacteria;c__Gammaproteobacteria	1.10%	3.20%	11.40%	3.90%	10.50%	6.50%	3.80%	3.00%	11.70%	0.90%	8.40%	3.50%	1.10%	2.80%	2.60%	2.60%
k__Bacteria;p__Spirochaetes;c__Spirochaetes	0.00%	0.00%	0.50%	0.00%	0.00%	0.00%	0.00%	0.00%	0.00%	0.00%	0.00%	0.00%	0.00%	0.00%	0.40%	0.00%
k__Bacteria;p__Spirochaetes;c__[Brachyspirae]	0.00%	0.00%	0.00%	0.00%	0.00%	0.00%	0.00%	0.00%	0.00%	0.00%	0.00%	0.00%	0.00%	0.00%	0.00%	0.20%
k__Bacteria;p__Synergistetes;c__Synergistia	0.00%	0.00%	0.00%	0.00%	0.00%	0.00%	0.00%	0.00%	0.00%	0.00%	0.00%	0.00%	0.00%	0.10%	0.10%	0.00%
k__Bacteria;p__TM7;c__TM7-3	6.60%	3.40%	0.40%	0.70%	5.00%	0.60%	0.10%	0.30%	0.20%	0.60%	0.20%	0.70%	0.30%	0.20%	0.00%	0.30%
k__Bacteria;p__Tenericutes;c__Mollicutes	0.60%	0.40%	0.30%	0.10%	0.30%	0.10%	0.20%	0.00%	0.00%	0.30%	0.10%	0.20%	0.10%	0.00%	0.00%	0.00%
k__Bacteria;p__Verrucomicrobia;c__Verrucomicrobiae	0.00%	1.50%	0.00%	0.00%	0.00%	0.00%	0.00%	0.00%	0.00%	0.00%	0.00%	0.00%	0.00%	1.80%	0.00%	0.00%
